# Liposomal Formulations to Improve Antioxidant Power of Myrtle Berry Extract for Potential Skin Application

**DOI:** 10.3390/pharmaceutics14050910

**Published:** 2022-04-21

**Authors:** Maria De Luca, Daniela Lucchesi, Carlo Ignazio Giovanni Tuberoso, Xavier Fernàndez-Busquets, Antonio Vassallo, Giuseppe Martelli, Anna Maria Fadda, Laura Pucci, Carla Caddeo

**Affiliations:** 1 Department of Scienze, University of Basilicata, Viale dell’Ateneo Lucano 10, 85100 Potenza, Italy; maria.deluca@unibas.it (M.D.L.); antonio.vassallo@unibas.it (A.V.); giuseppe.martelli@unibas.it (G.M.); 2 KAMABIO Srl, Via Al Boschetto 4/B, 39100 Bolzano, Italy; 3 Section of Diabetes and Metabolic Diseases, Department of Clinical and Experimental Medicine, University of Pisa, Via Piero Trivella, 56124 Pisa, Italy; d.lucchesi@ao-pisa.toscana.it; 4 Department of Scienze della Vita e dell’Ambiente, University of Cagliari, S.P. Monserrato-Sestu Km 0.700, 09042 Monserrato, Italy; tuberoso@unica.it; 5 Nanomalaria Group, Institute for Bioengineering of Catalonia (IBEC), The Barcelona Institute of Science and Technology, Baldiri Reixac 10-12, 08028 Barcelona, Spain; xfernandez_busquets@ub.edu; 6 Barcelona Institute for Global Health (ISGlobal), Hospital Clínic-Universitat de Barcelona, 08036 Barcelona, Spain; 7 Spinoff TNcKILLERS Srl, Viale dell’Ateneo Lucano 10, 85100 Potenza, Italy; 8 Department of Scienze della Vita e dell’Ambiente, Sezione di Scienze del Farmaco, University of Cagliari, Via Ospedale 72, 09124 Cagliari, Italy; mfadda@unica.it; 9 Institute of Agricultural Biology and Biotechnology, CNR Pisa, Via Moruzzi 1, 56124 Pisa, Italy

**Keywords:** myrtle extract, liposomes, antioxidant, fibroblast, skin

## Abstract

Many substances in plant extracts are known for their biological activities. These substances act in different ways, exerting overall protective effects against many diseases, especially skin disorders. However, plant extracts’ health benefits are often limited by low bioavailability. To overcome these limitations, drug delivery systems can be employed. In this study, we evaluated the antioxidant power of an ethanolic extract from *Myrtus communis* L. (myrtle) berries through colorimetric tests (DPPH and FRAP). The antioxidant activity was also verified by using fibroblast cell culture through cellular Reactive Oxygen Species (ROS) levels measurements. Moreover, the myrtle extract was formulated in phospholipid vesicles to improve its bioavailability and applicability. Myrtle liposomes were characterized by size, surface charge, storage stability, and entrapment efficiency; visualized by using cryo-TEM images; and assayed for cytocompatibility and anti-ROS activity. Our results suggest that myrtle liposomes were cytocompatible and improved the extract’s antioxidant power in fibroblasts, suggesting a potential skin application for these formulations and confirming that nanotechnologies could be a valid tool to enhance plant extracts’ potentialities.

## 1. Introduction

*Myrtus communis* L., commonly referred to as myrtle, is an evergreen shrub belonging to the Myrtaceae family. The plant presents a stem up to three meters tall, small green leaves, light-colored flowers, and round blue-black berries that are extremely rich in seeds. Myrtle is widely distributed in the Mediterranean area, and different parts of the plant are traditionally used for many purposes. The berries and leaves are used as flavoring agents and in the production of a liqueur typical to Sardinia (Italy) [1,2,3,4]. Other uses for myrtle include animal feed and cosmetic and pharmaceutical applications.

Berry extracts, as well as seed extracts, are known for various biological activities [5,6,7,8,9,10,11,12]. The broad spectrum of biological activities associated with myrtle is ascribed to many isolated components. Phytochemical analysis has revealed its richness in phenolic compounds, including phenolic acids, flavan-3-ols, and anthocyanins. Moreover, essential oils and polyunsaturated fatty acids have been detected [13,14,15,16,17,18]. One of berry extracts’ best-known actions is their antioxidant effects [19]. Some studies have highlighted their inhibitory activity against enzymes linked to neurodegenerative diseases [20], beneficial effects on gastrointestinal disorders [21,22,23], ability to reduce streptozotocin-induced oxidative stress in diabetic rats [24], and wound-healing properties [25]. These properties can all be related to myrtle extract’s antioxidant activity.

Given the above, myrtle is a potential source of antioxidants with beneficial health effects, especially against skin disorders. Antioxidants’ main action for skin health is fighting oxidative stress caused by free radicals. However, the benefits provided by natural antioxidants can be limited by their low bioavailability in biological systems. Drug delivery systems may overcome these limitations by increasing bioavailability; preventing degradation of sensible compounds; and offering a controlled rate of release, increased efficacy, and reduced toxicity [26,27,28].

In this study, an ethanolic myrtle berry extract was produced, characterized by liquid chromatography, and formulated in liposomes intended for skin application. The phospholipid vesicles were characterized by light scattering for size, surface charge, and storage stability. Their morphology was observed under cryo-TEM, and the entrapment efficiency was evaluated via HPLC-quantification of major phenolic compounds. The antioxidant power of the formulations was assayed by colorimetric tests (DPPH and FRAP) and by in vitro analyses through ROS levels measurements on fibroblast cells.

## 2. Materials and Methods

### 2.1. Materials

Lipoid S75 (fat-free soybean phospholipids with 70% phosphatidylcholine; S75) was supplied by Lipoid GmbH (Ludwigshafen, Germany). Standards of phenolic compounds (cyanidin-3-*O*-glucoside, delphinidin-3-*O*-glucoside, malvidin-3-*O*-glucoside, peonidin-3-*O*-glucoside, petunidin-3-*O*-glucoside, gallic acid, ellagic acid, myricetin-3-*O*-galactoside, and myricetin-3-*O*-rhamnoside) were purchased from Extrasynthese (Genay Cedex, France). Ultrapure water (18 MΩ cm) was obtained with a Milli-Q Advantage A10 System apparatus (Millipore, Milan, Italy). Acetonitrile for ultra-phase liquid chromatography (UPLC, gradient grade) analyses, methanol, phosphoric acid, phosphate buffered saline (PBS), 2,2-diphenyl-1-picrylhydrazyl (DPPH), 6-hydroxy-2,5,7,8-tetramethylchroman-2-carboxylic acid (Trolox), 2,4,6-tris(pyridin-2-yl)-1,3,5-triazine (TPTZ), 3-(4,5-dimethylthiazol-2-yl)-2,5-diphenyltetrazolium bromide (MTT), and other reagents were purchased from Sigma-Aldrich/Merck (Milan, Italy), unless otherwise specified.

### 2.2. Plant Material and Extract Preparation

Purple myrtle (*Myrtus communis* L.) berries were randomly collected in Monte Arcosu (Sardinia, Italy) in December 2020. The berries (1 kg) were gently cleaned before being ground in a mortar, macerated with 1 L of ethanol 96% (1:1, *w/v*), and incubated twice for 30 min under sonication at 15 ± 2 °C. The supernatant was separated, and the berries were manually pressed to recover more liquid. Afterward, 1 L of ethanol 96% was poured on the exhausted berries, and the procedure was repeated. The supernatants were joined, filtrated using a strainer, and then concentrated by vacuum distillation (Büchi Rotavapor R-114, Flawil, Switzerland) until the alcohol was completely eliminated. The obtained extract was stored at −20 °C and later used for liposome preparation.

### 2.3. HPLC–DAD Analysis

The identification and quantification of phenolic compounds in the myrtle berry extract was performed by HPLC–DAD analysis, as described by Montoro et al. [29]. A 1260 Infinity II HPLC system (Agilent Technologies, Cernusco sul Naviglio, Milan, Italy) with a G7111A pump, a G7129A autosampler, and a G4212B photodiode array detector was used. A Kinetex EVO C18 column (150 × 4.6 mm, 2.6 μm, Phenomenex, Casalecchio di Reno, Bologna, Italy) was used, with a mobile phase consisting of 0.22 M phosphoric acid (solvent A) and acetonitrile (solvent B) delivered at a constant flow rate of 0.8 mL/min, following a gradient elution: from 100% solvent A to 80% solvent A in 20 min, to 70% in 35 min, to 0% in 45 min, and kept stable for up to 50 min. Then, solvent A was brought back to 100% and kept stable for 5 min before injection. The injection volume was 10 μL. The chromatograms and spectra were elaborated with an OpenLab V. 2.51 data system (Agilent Technologies), and polyphenols were detected and quantified according to the main classes: anthocyanins at 520 nm, flavonols at 360 nm, and hydroxybenzoic acids at 280 nm. The standards of phenolic compounds were dissolved in methanol and the working solutions were prepared in ultrapure water. The calibration curves were plotted by using the external standard method: the peak area was correlated with the concentration by means of the least-squares method, with a coefficient of determination (r^2^) > 0.999 in the range of 10–1000 µg/L for all the compounds. Individual components were identified by comparing the retention time and UV–VIS spectra of pure commercial standards. For the HPLC–DAD analysis, myrtle berry extract was diluted 1:200 *w/v* with a methanol: 0.22 M phosphoric acid 80:20 *v/v* mixture, and liposomes were injected after dilution with methanol (1:100 *v/v*; see Section 2.4). The solutions were filtered with a 0.45 μm cellulose acetate syringe filter before injection.

### 2.4. Vesicle Preparation and Characterization

For liposomes preparation, myrtle berry extract and S75 were dispersed in PBS and sonicated (10 cycles of 5 s on/2 s off + 5 cycles 2 s on/2 s off; 13 µm of probe amplitude) with a Soniprep 150 disintegrator (MSE Crowley, London, UK).

For a proper comparison, empty liposomes were prepared according to the same procedure as myrtle liposomes, but without the myrtle extract (Table 1).

The formation of the vesicles was confirmed by cryogenic-transmission electron microscopy (cryo-TEM), which also provided information about their morphology. The vesicle dispersion (5 µL) was placed on a glow-discharged holey carbon grid and then blotted against filter paper. The obtained thin film was vitrified by plunging the grid (100% humidity, room temperature) into ethane kept at its melting point with liquid nitrogen, using a Vitrobot (FEI Company, Eindhoven, The Netherlands). The vitrified film was transferred to a Tecnai F20 TEM (FEI Company) by using a cryo-transfer (Gatan, Pleasanton, CA, USA) for sample observation. Images were acquired at 200 kV and at –170/–175 °C, using low-dose imaging conditions not exceeding 20 e^–^/Å^2^, with a 4096 × 4096 pixel CCD Eagle camera (FEI Company).

The average diameter, polydispersity index, and zeta potential of the phospholipid vesicles were measured by dynamic and electrophoretic light-scattering techniques. The dispersions were properly diluted with PBS and analyzed by using a Zetasizer nano-ZS (Malvern Panalytical, Worcestershire, UK).

The non-incorporated extract components were removed from the vesicle dispersions through dialysis. A 1 mL sample was loaded into Spectra/Por^®^ tubing (12,000–14,000 Da MWCO; Spectrum, DG Breda, The Netherlands) and kept in water (2 L), under gentle stirring, for 2 h. Non-dialyzed and dialyzed vesicles were disrupted by diluting (1:100) with methanol and analyzed by HPLC–DAD analysis (see Section 2.3) to determine the amounts of targeted phenolic compounds. The entrapment efficiency (EE) was calculated as the percentage of the phenolic compounds detected in dialyzed vs. non-dialyzed myrtle liposomes.

### 2.5. Antioxidant Assays

The DPPH assay was carried out to evaluate the antioxidant activity of myrtle extract (20 mg/mL) in aqueous solution or in liposomes. Empty liposomes were also tested to evaluate the activity of the nanosystem. Each sample (10 µL) was added to a DPPH methanolic solution (25 µM; 2 mL) and incubated at room temperature in the dark for 30 min. Discoloration of the DPPH solution occurs as a function of the antioxidant power and the concentration of a sample, leading to a decrease in absorbance (*A*) at 517 nm. The antioxidant activity (*AA*) of the samples was calculated according to Equation (1) [30]:

(1) $$AA=\left(\frac{A_{DPPH}\;-\;A_{sample}}{A_{DPPH}}\right)\times \;100$$

The results were expressed also as Trolox Equivalents (TEs). The TE values (µg TE/mL solution) were calculated by using a calibration curve (Trolox concentration range: 0–500 µg/mL). The IC_50_ (mg/mL), the concentration required to cause 50% DPPH inhibition, was calculated by interpolation of linear regression equation obtained by plotting *AA* against a range of myrtle extract’s concentrations (20−0.625 mg/mL).

The antioxidant activity of myrtle extract (20 mg/mL), either in aqueous solution or in liposomes, and empty liposomes was assessed also by the FRAP (ferric reducing antioxidant power) assay, which is based on the reduction of Fe^3+^-TPTZ to Fe^2+^-TPTZ that causes an increase in absorbance [31]. A total of 10 µL of each sample was mixed with 2 mL of the TPTZ–ferric solution. After 4 min of incubation at room temperature in the dark, the absorbance was read at 593 nm. The results, expressed as µg Fe^2+^ equivalents/mL solution, were calculated by using a calibration curve (FeSO_4_ concentration range: 13.9−2317 µg/mL). The EC_50_ (mg/mL), the concentration required to increase FRAP absorbance by 50%, was calculated by interpolation of linear regression equation obtained by plotting absorbance against a range of myrtle extract’s concentrations (20−0.625 mg/mL).

### 2.6. Fibroblast Cell Culture

The 3T3-L1 cells (ATCC^®^ CL-173^TM^; Manassas, VA, USA) were grown in Dulbecco’s modified Eagle’s medium (DMEM; EuroClone S.p.A., Pero, Milan, Italy) supplemented with 10% fetal bovine serum (FBS; Sigma-Aldrich Inc., St. Louis, MO, USA), 100 units/mL penicillin (Life Technologies, Carlsbad, CA, USA), and 100 μg/mL streptomycin (Life Technologies) and maintained at 37 °C in a humidified 5% CO_2_ incubator. The cells were seeded into 96-well plates and tested under the following experimental conditions:

- Cells unexposed (negative control) or exposed to 500 μM 2,2′-azobis(2-methylpropionamidine) dihydrochloride (AAPH; Fluka-Sigma-Aldrich Inc., St. Louis, MO, USA), a peroxyl radical generator used as a positive control, for 4 and 23 h;
- Cells exposed to myrtle aqueous solution or myrtle liposomes, previously diluted to reach the required doses of myrtle (0.1, 1.0 and 10 μg/well), for 5 and 24 h;
- Cells exposed to myrtle aqueous solution or myrtle liposomes, previously diluted to reach the required dose of myrtle (10 μg/well), for 1 h and co-incubated with 500 μM AAPH for a further 4 h.

For comparative purposes, empty liposomes were tested at the same dilutions as the myrtle solution or liposomes.

### 2.7. Assessment of Viability

The viability of 3T3-L1 cells was assessed by the MTT assay after treatments listed in Section 2.6. The MTT assay relies on the mitochondrial activity of live cells to convert yellow MTT reagent into purple formazan, detectable via spectrophotometry. The fibroblasts (5 × 10^4^ cells/well) were incubated with MTT (0.5 mg/mL) for 3 h at 37 °C. The medium was removed, and a DMSO:isopropanol (10:90, *v/v*) mixture was added to the cells. The dye released from the cells was quantified by reading the absorbance at 540 nm (reference wavelength: 620 nm) with a Multiskan EX multiplate reader (ThermoFisher Scientific, Waltham, MA, USA). The experiment was performed at least three times independently, each time in triplicate.

### 2.8. Assessment of Cellular Reactive Oxygen Species (ROS) and Cell Morphology

The 3T3-L1 cells were incubated with the samples alone or the samples and 500 μM AAPH, and the endogenous or chemically induced cellular ROS were detected. The fibroblasts were seeded into a 96-well blackened fluorescence plate (5 × 10^4^ cells/well) and incubated with 5-(and-6)-chloromethyl-2′,7′-dichlorodihydrofluorescein diacetate, acetyl ester (CM-H_2_DCF-DA; Invitrogen, ThermoFisher Scientific, Waltham, MA, USA) (5 μM/well) for 60 min, at 37 °C, in the dark. The cells were rinsed with 1× PBS to remove CM-H_2_DCF-DA and treated according to the experimental conditions reported in Section 2.6. ROS production was detected by measuring the increase in fluorescence with a microplate reader. Fluorescence was measured by excitation at 495 nm and emission at 527 nm, using a Varian Cary Eclipse Spectrophotometer (Variant/Agilent Technologies, Santa Clara, CA, USA). The experiment was repeated at least three times independently, each time in quadruplicate.

To assess cell morphology, 3T3-L1 cells, untreated or incubated with 500 μM AAPH, or co-incubated with 500 μM AAPH and myrtle aqueous solution, empty or myrtle liposomes for 5 h were examined under a Primo Vert inverted microscope (Carl Zeiss Microscopy GmbH, Jena, Germany).

### 2.9. Statistical Analysis of Data

Results are expressed as means ± standard deviations (SDs). One-way analysis of variance (ANOVA) was used to determine statistically significant differences between the means of independent groups, and Fisher’s post hoc test was used for single comparisons. The *p*-values below 0.05 were considered statistically significant. StatView software package (SAS Institute Inc., Cary, NC, USA) was used.

## 3. Results

### 3.1. Quantification of Phenolic Compounds

**Figure 1:**
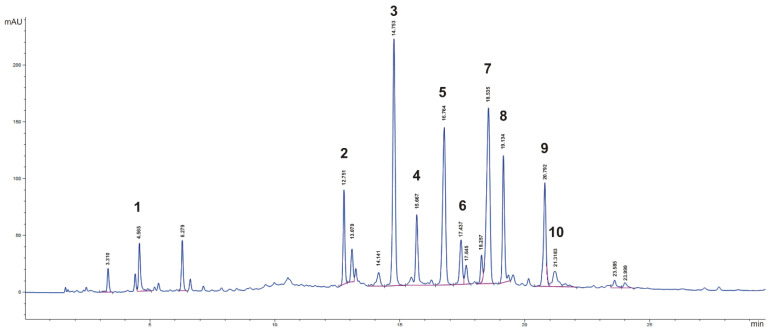
HPLC–DAD chromatogram of myrtle berry extract at λ = 280 nm. Chromatographic conditions are described in the text. 1: Gallic acid; 2: Gallic acid derivative; 3: Delphinidin-3-*O*-glucoside; 4: Cyanidin-3-*O*-glucoside; 5: Petunidin-3-*O*-glucoside; 6: Peonidin-3-*O*-glucoside; 7: Malvidin-3-*O*-glucoside; 8: Myricetin-3-*O*-galactoside; 9: Myricetin-3-*O*-rhamnoside; 10: Ellagic acid.

The HPLC–DAD qualitative evaluation of the major phenolic compounds in the myrtle extract showed the purple myrtle berries’ typical composition [19,32]. The most abundant polyphenolic compounds were anthocyanins (delphinidin, cyanidin, malvidin 3-*O*-glucosides, petunidin-3-*O*-glucoside, and peonidin-3-*O*-glucoside: 122.41 ± 0.1, 81.81 ± 1.6, 139.41 ± 1.4, 115.01 ± 1.6, and 84.61 ± 0.4 µg/L, respectively) and two flavonoids, myricetin derivatives (myricetin-3-*O*-galactoside and myricetin-3-*O*-rhamnoside: 208.6 ± 2.1 and 207.4 ± 3.3 µg/L, respectively) (Figure 1). Ellagic acid was also detected in the chromatogram at 280 nm (17.1 ± 1.2 µg/L), along with several galloyl derivatives, including gallic acid (87.5 ± 6.8 µg/L).

### 3.2. Vesicle Design and Characterization

This study’s aim was to develop a stable liposomal formulation of a myrtle extract and demonstrate that it was safe for cells and could improve the extract’s bioactive potential in vitro. Particularly, a potential topical application of this formulation for the treatment of oxidative-stress-related disorders is supposed.

The liposomes were characterized in terms of mean diameter, polydispersity, and zeta potential. To evaluate the myrtle extract’s impact on the vesicles’ characteristics, the liposomes with myrtle extract added were compared with the empty liposomes. The light-scattering results, as reported in Table 2, showed that the empty liposomes were 95 nm in diameter, monodispersed (PI 0.20), and negatively charged (−10 mV). The extract’s loading significantly increased the vesicles’ mean diameter, although they remained small (around 100 nm), whereas the polydispersity index and zeta potential values were unaltered (Table 2).

**Figure 2:**
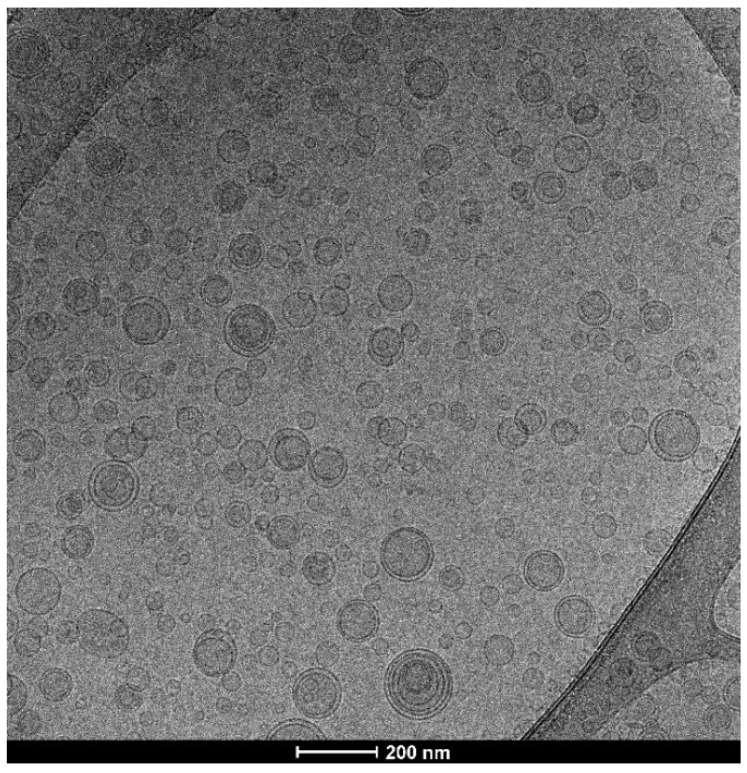
Myrtle liposomes through cryo-TEM observation.

The formation of vesicular structures characterized by their small size was confirmed by cryo-TEM observation. Figure 2 shows spherical oligolamellar vesicles at around 100 nm in diameter, which aligns with the light-scattering data.

The stability of the liposomal formulations was evaluated by monitoring the mean diameter, the polydispersity index, and the zeta potential during storage at 4 °C. No signs of significant alterations were detected.

The entrapment efficiency of the liposomes was calculated based on the amount of 10 targeted phenolic compounds identified in the myrtle extract and detected in the dialyzed and non-dialyzed dispersions. The liposomes entrapped high amounts of extract; the entrapment efficiency was at least 71.4% (for myricetin-3-*O*-galactoside) and over 95% for most of the anthocyanins (Table 3).

### 3.3. Antioxidant Assays

The antioxidant activity of the myrtle formulations was estimated as a function of their radical scavenging and ferric-reducing abilities (Table 4). The myrtle solution scavenged the DPPH radical completely (AA 96%), corresponding to 344 μg/mL of Trolox equivalents, and the IC_50_ value was 3.2 ± 0.58 mg/mL. Given the presence of phosphatidylcholine, empty vesicles possess a slight antioxidant activity (AA 39%). The level of antioxidant activity for the myrtle liposomes was slightly lower than the myrtle solution, with a statistically significant difference. Nevertheless, the antioxidant activity was greater than 90%, corresponding to 326 μg/mL of Trolox equivalents (Table 4), with an IC_50_ value of 3.3 ± 0.06 mg/mL.

The results of the FRAP assay showed that the myrtle solution had a strong reducing power of 1867 μg/mL of ferrous equivalents, with an EC_50_ value of 9.2 ± 0.85 mg/mL. The myrtle liposomes displayed similar values, with no statistically significant differences. In addition, the empty liposomes showed a slight reducing power (Table 4).

These findings confirm that the antioxidant activity of the myrtle extract was retained in the vesicle formulation.

### 3.4. Cell Viability and Anti-ROS Activity

**Figure 3:**
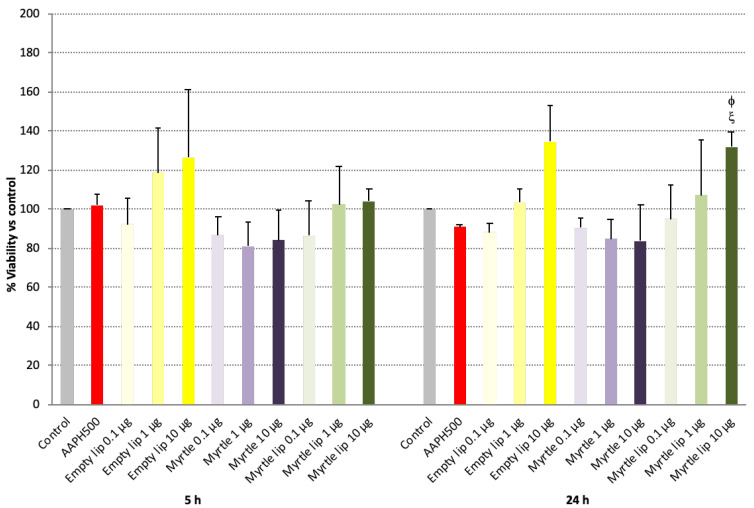
Viability of 3T3-L1 cells upon exposure to empty liposomes, myrtle solution, and myrtle liposomes for 5 and 24 h. Data are expressed as means ± standard error (SE); *n* = 3; ^φ^ *p* < 0.05 vs. myrtle solution 1 μg; ^ξ^ *p* < 0.05 vs. myrtle solution 10 μg.

The absence of cytotoxic effects of the liposome formulations was evaluated by using 3T3-L1 fibroblasts for viability after 5 and 24 h of exposure to increasing doses of myrtle extract (Figure 3). After 5 h, slight cytotoxicity (~10–20% mortality) was induced with all doses of the myrtle solution, even though it was not statistically significant compared with the untreated control cells. In contrast, this effect was not detected in the cells treated with myrtle liposomes, which appeared to prevent the inner toxicity of the myrtle extract.

The same trend was observed after 24 h of treatments, confirming the positive impact of the nanoformulation.

**Figure 4:**
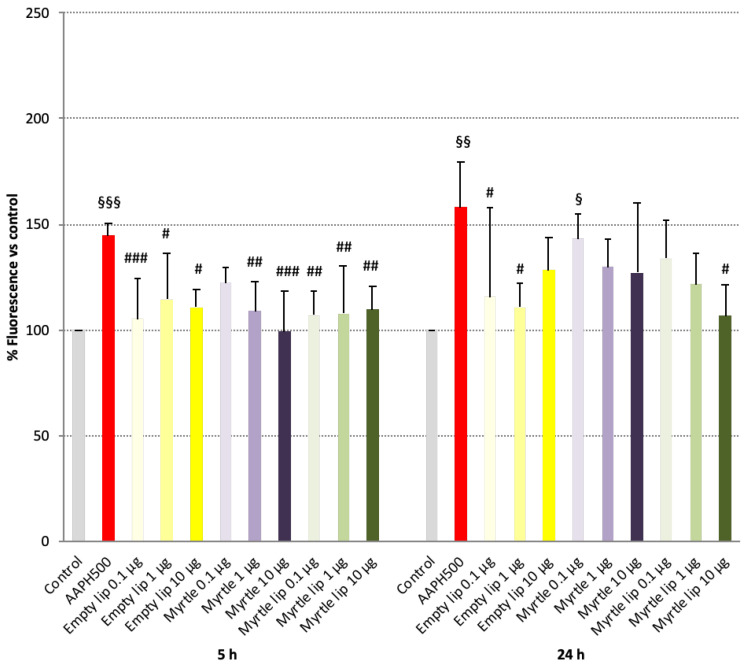
Effects of 500 µM AAPH, empty liposomes, myrtle solution, and myrtle liposomes on ROS production in 3T3-L1 cells after 5 and 24 h of incubation. Data are expressed as means ± SD; *n* = 3. ^#^ *p* < 0.05 vs. 500 µM AAPH; ^##^ *p* < 0.01 vs. 500 µM AAPH; ^###^ *p* < 0.005 vs. 500 µM AAPH; ^§^ *p* < 0.05 vs. control (i.e., cells without AAPH); ^§§^ *p* < 0.01 vs. control; ^§§§^ *p* < 0.005 vs. control.

The safety of the myrtle liposomes was also assessed as the absence of endogenous ROS production. As shown in Figure 4, after 5 h of exposure to the formulations of fibroblasts, none of them induced the formation of free radicals. The values were statistically similar (*p* > 0.05) to those of the untreated control cells and statistically different (*p* < 0.05−0.005) from cells treated with AAPH, a known radical generator. It must be pointed out that 500 μM of AAPH induced a significant increase in ROS levels, without affecting the viability of the cells compared to the control (Figure 3 and Figure 4). After 24 h of exposure, the myrtle solution triggered ROS production to a slight extent, yet statistically different from control levels (*p* < 0.05), and this effect was mitigated by the incorporation in liposomes (Figure 4).

**Figure 5:**
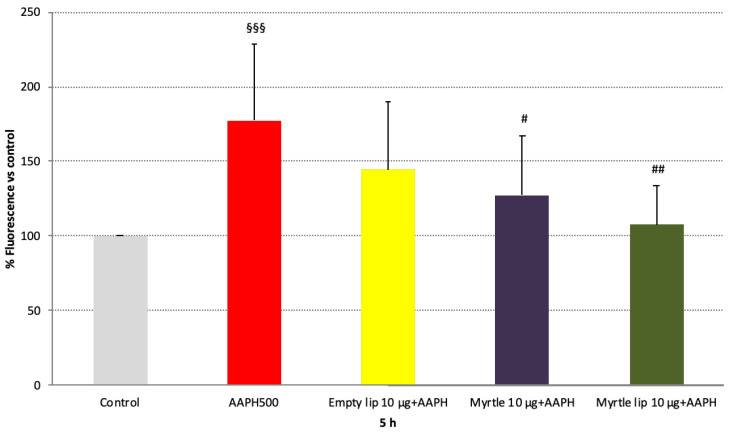
Anti-ROS effect of empty liposomes, myrtle solution, and myrtle liposomes on 3T3-L1 cells stressed with AAPH (500 µM). Data are expressed as means ± SD; n = 3. ^§§§^ *p* < 0.005 vs. control (i.e., cells without AAPH); ^#^ *p* < 0.05 vs. 500 µM AAPH; ^##^ *p* < 0.01 vs. 500 µM AAPH.

The antioxidant activity of the myrtle formulations was analyzed in AAPH-stressed fibroblasts as a function of their ability to reduce ROS levels, using DCFH-DA, a cell-permeable dye sensitive to the cellular redox state. In light of the results reported above on cell viability and endogenous ROS production, the higher dose of myrtle was evaluated (Figure 5).

After 5 h of treatment with the myrtle solution, a marked decrease in AAPH-induced ROS levels was detected (*p* < 0.05 vs. 500 µM AAPH). When the myrtle extract was delivered using liposomes, a further reduction was apparent (*p* < 0.01 vs. 500 µM AAPH), and the basal ROS levels were restored (*p* = ns vs. control). Furthermore, the results displayed the contribution provided by the nanosystem to the antioxidant activity of the myrtle formulation. Given their phospholipid content, empty liposomes exerted a minimum anti-ROS effect (Figure 5), and their carrier capabilities facilitated the transport of the myrtle bioactive compounds through the cell membrane. This resulted in a superior efficacy of the liposomal formulation.

**Figure 6:**
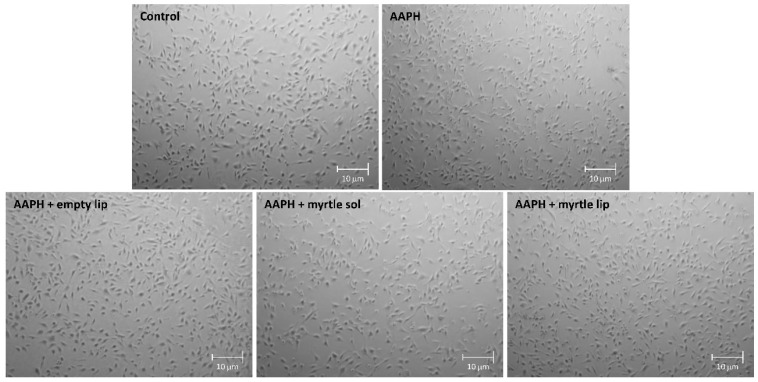
Representative microscope images of untreated 3T3-L1 cells in comparison with cells stressed with 500 µM AAPH or stressed with AAPH and treated with empty liposomes, myrtle solution, and myrtle liposomes for 5 h.

The evaluation of cell morphology confirmed these results. Figure 6 shows a slight reduction in the number of cells in cells treated with the myrtle solution, similar to what was observed in AAPH-stressed cells. In contrast, the AAPH-stressed cells treated with myrtle liposomes displayed features similar to non-stressed control cells.

## 4. Discussion

The encapsulation of natural bioactive substances in phospholipid vesicles is one of the most promising methods to overcome obstacles related to undesirable properties of natural extracts, such as low solubility, high instability, and low bioavailability. Nanotechnologies improve the release profile, absorption, distribution, and bioavailability of bioactive compounds and stabilize them against a wide range of environmental and chemical factors.

Based on our research, only one study on myrtle extract in vesicle formulation was reported. Despite this, the extract was obtained from myrtle leaves [33]. In our study, liposomes were prepared by using a simple and fast method based on the sonication of phospholipids and the myrtle berry extract in the dispersant solution. The obtained liposomes were characterized by their smaller size and higher entrapment efficiency, improving the formulation’s potential.

The high antioxidant activity of the myrtle berry extract was proved in numerous studies documented in the literature. However, none of them assessed the retention of such activity after the nanoformulation process. Snoussi et al. [34] found that the antioxidant activity of myrtle berry extract, as evaluated by the DPPH assay, ranged from 67% to 87.5%, depending on the amount of ethanol (60–90%) in the extraction mixture. Polat et al. [35] found that the antioxidant activity of myrtle berry extract, as evaluated by the DPPH assay, ranged from 6.73% to 65.6%, depending on the extracting solvents (70% acetone:water (*v/v*), 80% ethanol:water (*v/v*), 80% methanol:water (*v/v*), and distilled water). Hence, different results can be obtained depending on the processing conditions. Moreover, the extracts are complex mixtures that could also contribute to diverse results.

As shown by the DPPH assay, the myrtle extract showed prominent antioxidant activity (96%) retained after the nanoformulation and confirmed by the FRAP assay. The nanoformulation had the primary task of increasing myrtle bioavailability in cells and limiting potential toxicity without hindering its antioxidant action. The results obtained in fibroblasts demonstrated that the liposome formulation did not stress the cells and protected them from chemically induced oxidative stress more efficiently than the free myrtle extract.

## 5. Conclusions

The encapsulation techniques used in this study proved to be helpful in producing small stable liposomes with a high entrapment efficiency, without interfering with the antioxidant properties of the myrtle berry extract. Colorimetric tests and ROS-level measurements in fibroblasts confirmed the high antioxidant activity of the myrtle extract, and it was even greater after the liposomal nanoformulation. Moreover, the in vitro analyses of fibroblasts demonstrated the safety of myrtle liposomes at the tested doses. These results are consistent with this study’s aim to develop a safe, effective, and usable formulation for a topical application that can prevent oxidative-stress-related skin disorders.

## Figures and Tables

**Table 1:** Composition of the liposome formulations.

Formulation	S75	Myrtle Extract	PBS
Empty liposomes	180 mg		1 mL
Myrtle liposomes	180 mg	20 mg	1 mL

**Table 2:** Characteristics of empty and myrtle liposomes: mean diameter (MD), polydispersity index (PI), and zeta potential (ZP). Each value represents the mean ± SD (*n* > 10). ** Values statistically different (*p* < 0.01) from empty liposomes.

Formulation	MD nm ± SD	PI ± SD	ZP mV ± SD
Empty liposomes	95 ± 4.6	0.20 ± 0.03	−10 ± 1.1
Myrtle liposomes	** 102 ± 5.6	0.22 ± 0.02	−10 ± 0.8

**Table 3:** Entrapment efficiency (E%) of the main phenolic compounds identified in myrtle extract.

No.	Compound	E%
1	Gallic acid	90.4 ± 0.7
2	Gallic acid derivative *	89.7 ± 1.2
3	Delphinidin-3-*O*-glucoside	95.4 ± 0.6
4	Cyanidin-3-*O*-glucoside	96.2 ± 0.1
5	Petunidin-3-*O*-glucoside	96.8 ± 1.4
6	Peonidin-3-*O*-glucoside	96.9 ± 1.1
7	Malvidin-3-*O*-glucoside	85.5 ± 4.3
8	Myricetin-3-*O*-galactoside	71.4 ± 2.3
9	Myricetin-3-*O*-rhamnoside	84.0 ± 4.4
10	Ellagic acid	78.7 ± 5.3

* Dosed with the calibration curve for gallic acid. Data are given as the mean ± SD (*n* = 4).

**Table 4:** In vitro antioxidant activity of myrtle formulations. For the DPPH assay, results are expressed as AA (%) and TE (μg Trolox equivalents/mL solution); for the FRAP assay, results are expressed as FE (µg Fe^2+^ equivalents/mL solution). Results are reported as the mean ± SD of at least three separate experiments, each performed in triplicate. ** Statistically different values (*p* < 0.01) from the myrtle solution.

Formulation	DPPH Assay		FRAP Assay
	AA (%)	TE (µg Trolox Equivalents/mL)	FE (µg Fe^2+^ Equivalents/mL)
Myrtle solution	96 ± 1.4	344 ± 22	1867 ± 32
Empty liposomes	39 ± 7.4	137 ± 19	602 ± 46
Myrtle liposomes	** 91 ± 0.8	326 ± 17	1831 ± 70

## Data Availability

The data presented in this study are available within this article.
